# Emotion detection using electroencephalography signals and a zero-time windowing-based epoch estimation and relevant electrode identification

**DOI:** 10.1038/s41598-021-86345-5

**Published:** 2021-03-29

**Authors:** Sofien Gannouni, Arwa Aledaily, Kais Belwafi, Hatim Aboalsamh

**Affiliations:** Computer Science Department, College of Computer and Information Sciences, King Saud University, Riyadh, 11543 Saudi Arabia

**Keywords:** Computational biology and bioinformatics, Neuroscience

## Abstract

Recognizing emotions using biological brain signals requires accurate and efficient signal processing and feature extraction methods. Existing methods use several techniques to extract useful features from a fixed number of electroencephalography (EEG) channels. The primary objective of this study was to improve the performance of emotion recognition using brain signals by applying a novel and adaptive channel selection method that acknowledges that brain activity has a unique behavior that differs from one person to another and one emotional state to another. Moreover, we propose identifying epochs, which are the instants at which excitation is maximum, during the emotion to improve the system’s accuracy. We used the zero-time windowing method to extract instantaneous spectral information using the numerator group-delay function to accurately detect the epochs in each emotional state. Different classification scheme were defined using QDC and RNN and evaluated using the DEAP database. The experimental results showed that the proposed method is highly competitive compared with existing studies of multi-class emotion recognition. The average accuracy rate exceeded 89%. Compared with existing algorithms dealing with 9 emotions, the proposed method enhanced the accuracy rate by 8%. Moreover, experiment shows that the proposed system outperforms similar approaches discriminating between 3 and 4 emotions only. We also found that the proposed method works well, even when applying conventional classification algorithms.

## Introduction

Affective Computing has emerged as an important field of study that aims to develop systems that can automatically recognize emotions. In 2019, the market for emotion-detection technology is worth roughly $21.6bn, and its value is predicted to reaching 56bn$ by 2024^1^. Emotions are human responses to environmental objects or events^2^ that affect different aspects of human life, such as attention, memorization, achieving goals, awareness of priority, knowledge motivation, communication with others, learning development, mood status, and effort motivation^3,4^. During the last decade, many research and development efforts have been deployed to develop new approaches and techniques for emotion recognition. Two main approaches have evolved the way researchers analyze and classify emotions: the constructionist and locationist approaches. The first approach defines several dimensions to create an effective framework for studying and classifying emotions. The valence-arousal-dominance (VAD) descriptive model^2,4^ is the model most representative of this approach. Conversely, the second approach assumes that there is a specific brain structure and pattern for each emotion.

It is becoming increasingly attractive to detect human emotions using biological brain signals. Electroencephalography (EEG) is a reliable and cost effective technology used to measure brain activity. Detecting emotion using EEG signals involves multiple steps being performed in sequence to satisfy the requirements of a brain–computer interface (BCI). Traditionally, these steps include removing artifacts from EEG signals, extracting temporal or spectral features from the EEG signal’s time or frequency domain, respectively, and finally, designing a multi-class classification strategy. Feature quality dramatically increases the accuracy of the emotion classification strategy.

Traditional methods are used to extract features from a fixed group of the same EEG channels for all subjects. However, brain-behavior is sophisticated and changes from one person to another^3^ and from one emotional state to another^5–7^. Moreover, extracted features are either computed from the whole sample of the EEG signal, which contains irrelevant information, or from an arbitrarily chosen portion of the sample and not necessarily the portion of the signal that corresponds to the emotional excitation instant. There is a growing need for additional steps, such as the identification of epochs, which are the instants at which excitation is maximum during the emotion, and the selection of electrodes that show significant variation in brain activity during emotional states, to accurately detect emotional states. Experiments show that the addition of these steps drastically improves the quality of features.

We propose a novel method for EEG channel selection based on signal epoch estimation using the zero-time windowing (ZTW) method^20^. The ZTW method was used to extract instantaneous spectral information from EEG signals at a good temporal resolution. The spectral information obtained using the Numerator Group Delay (NGD) function on the emotional EEG signal was analyzed using the ZTW method to identify the epoch locations on every EEG channel. A majority voting technique was used to select the location that was commonly identified as the epoch instant by most of the electrodes. Only EEG channels that had a vote that matched the selected epoch location were considered for further processing. The other channels were ignored. The brain activity of the selected channels was analyzed to determine which channels showed significant changes during each emotional state. The retained electrodes were used as sources to extract relevant features. The experimental results show that the proposed method is highly competitive compared with existing studies on multi-class emotion recognition. We also found that the proposed method works well, even when applying deep learning algorithms.

The motivation for this study is discussed in “Related works” section, and the most widely known research works in this area are mentioned. “Proposed method” section presents our approach to epoch identification, channel selection, and emotion recognition. “Performance evaluation of the proposed method” section describes the performance of our approach and compares it with related works. Finally, “Conclusion” section summarizes the study and discusses future perspectives of this work.

## Related works

Emotion recognition has gained substantial attention in the last decade because it is directly linked to psychology, physiology, learning studies, marketing, and healing. Previous studies have either used the emotion locationist or VAD descriptive models^2,4^. They have also tried different feature extraction methods and different feature types from the temporal domain, frequency domain, statistical features, and time-frequency features.

In^8^, the study proposed to recognize human emotions by combining six statistical parameters of EEG signals from the time domain as EEG features. The proposed approach performed a channel selection process to eliminate noisy and redundant channels using PCA and ReliefF algorithms. Then, SVM was trained and applied to identify emotions using the DEAP dataset. The experimental results achieved an average accuracy rate of 81.87%.

In the research undertaken in^9^, the researchers sought to reduce the feature vector dimension in order to improve the accuracy of the classification. Principal component analysis (PCA) was used to reduce the features. PCA was applied with SVM on the SEED dataset to classify positive, negative, and neutral emotions, and the reported accuracy rate was 85.85%. An important implication of these results is that a reduction of the feature dimension may lead to improved accuracy.

The accuracy of multi-class emotion detection systems has substantial room for improvement. The study in^10^ examined different types of classifiers on different feature types. The researchers extracted statistical features, power density, frequency-domain features, entropy, and wavelet energy. They chose 14 electrodes from the DEAP dataset and extracted up to 168 features for every subject. They applied KNN, SVM, and decision tree (DT) classifiers. The optimal result for valence and dominance was associated with the use of the KNN classifier with statistical EEG features. Specifically, valence amounted to 77.54%, and dominance amounted to 79%. For the same features, arousal was 78.88% using the SVM classifier.

Seminal contributions were made in^11^, which discussed the multi-class classification technique as a way to improve accuracy. At the outset, the researchers extracted multiple features from the time-domain of EEG signals, the frequency-domain, and time-frequency features. In turn, the researchers used a variant of the particle swarm optimization (PSO) algorithm for feature selection. This algorithm was improved using a multi-stage linearly decreasing inertia weight (MLDW). The researchers used SVM to classify four emotional states: high-arousal-high-valence, high-arousal-low-valence, low-arousal-high-valence, and low-arousal-low-valence. The average accuracy was 76.67%.

Liu et al., proposed a deep generalized canonical correlation analysis with an attention mechanism (DGCCA-AM), with emotion-related attention mechanism for feature fusion, to five-category emotion recognition^12^. A differential entropy (DE) features are extracted from EEG signals using a short-time Fourier transform (STFT) with 4 s nonoverlapping Hanning window. The proposed method was evaluated on a public multimodal dataset, SEED-V. The experimental results reached an accuracy of 82.11% for five emotion classifications with three modalities.

In^13^, Fuji et al. proposed a method to evaluate the degree of emotion being motivated in continuous music videos based on asymmetry index (AsI). The AsI is used to estimate the degree of emotional induction by measure the mutual information shared between two frontal lobes. After the calculation of the AsI, the signal segments with low emotional motivation is filtered, and effective emotional stimulus signals are reserved for feature extraction and emotion classification. A wavelet packet transform is used to gain wavelet packet coefficients, and the coefficients are clustered. The features were extracted from each sub-band and were sent to SVM for classification purposes. The obtained results reached an average recognition rate of 70.5%.

In^14^, Pane et al., proposed rule-based classifier and a decision tree algorithm to recognize emotions using EEG signals. They discriminate between four class target emotions (i.e., happy, angry, sad, and relax). This study considered different frequency bands of EEG signals. The band pass IIR filter with Chebyshev type II window was applied to separate the EEG signal into gamma, beta, alpha, and theta bands. Features were extracted from both the Time and frequency domains. The proposed rules classifier model generated 10 rules of emotion classification, while the validation of the rules achieved an average accuracy of 81.64% for relaxed emotion class using the DEAP database.

Several studies have applied deep learning to emotion recognition, and they have shown improved accuracy of emotion classification. A study in^15^ used DL to classify four emotional classes: angry, sad, happy, and relaxed. The researchers used multiple features, including time-domain, frequency-domain, and entropy values. ANN was applied to the three groups of features separately, and the DEAP dataset was used, including all 32 electrodes for feature extraction. Promising results in terms of accuracy were obtained, consisting of 93.48% for time-domain features, 92.44% for frequency-domain features, and 93.75% for entropy-based features (i.e., spectral entropy, Shannon entropy, and sample entropy).

Deep Learning in neural networks was continuing used in several studies. In study^16^, Two levels of valence and arousal were used in the research for emotion classification, and empirical mode decomposition (EMD) and multivariate EMD (MEMD) were used for emotion recognition. The method involved analyzing the non-linear and non-stationary time series of electroencephalography (EEG) signals. It decomposed the EEG signal into a set of segments, which are referred to as intrinsic mode functions (IMF). In turn, time-domain features and frequency-domain features were extracted. The researchers used an artificial neural network (ANN) classifier on the DEAP dataset, yielding an accuracy of 75% for arousal and 72.87% for valence.

In^17^, a recurrent neural network (RNN) was used for emotion recognition: emotions were classified by the level of valence, arousal, and liking from the VAD model. Long–short term memory (a special kind of RNN) was used with the structure of an input layer, four hidden layers, and one output layer. The activation functions used were ReLU and sigmoid, and a probability of 0.2 was used in the dropout layer and sigmoid in the dense layer. This model was applied to the DEAP dataset using all 32 EEG electrodes. The accuracy of the model was 85.65%, 85.45%, and 86.99% for arousal, valence, and liking, respectively.

In^18^, the researchers extracted the following short-time Fourier transform (STFT) features: from the original signal, as well as higher-order crossing (HOC) and Hilbert-Huang spectrum (HHS) from the alpha, beta, delta, and gamma bands. The researchers then eliminated the non-informative features using the mRMR algorithm. Finally, the researchers obtained 14 electrodes (AF3, F7, F3, FC5, T7, P7, O1, O2, P8, T8, FC6, F4, F8, and AF4) from the 32 electrodes in the DEAP dataset. LIBSVM was used to classify anger, surprise, and other. A one-vs-one classification approach was used to solve the multi-class problem. The results indicated that the use of mRMR improved the accuracy of the results, but the overall accuracy was lower than 60%.

In^19^, four deep belief network (DBN) models were presented to classify emotions using multimodal data including facial expressions, body gestures, vocal expressions and physiological signals. Experimental results show that the DBN models perform well for emotion recognition. The authors proposed convolutional deep belief network (CDBN) models that learn salient multimodal features of expressions of emotions. The average recognition accuracy of the proposed CDBN models achieved 79.2%.

This study proposes a new channel selection method that depends on the brain-behavior of the individual under study. The selection process was combined with the spectrum epoch selection using ZTW and NGD in the temporal domain. Then, the chosen channels were filtered with frequency power variation to select the strong electrodes in each emotional state, making the process of channel selection the core step in emotion classification using two algorithms: the quadratic discriminant classifier (QDC) and the RNN.

## Proposed method

**Figure 1:**
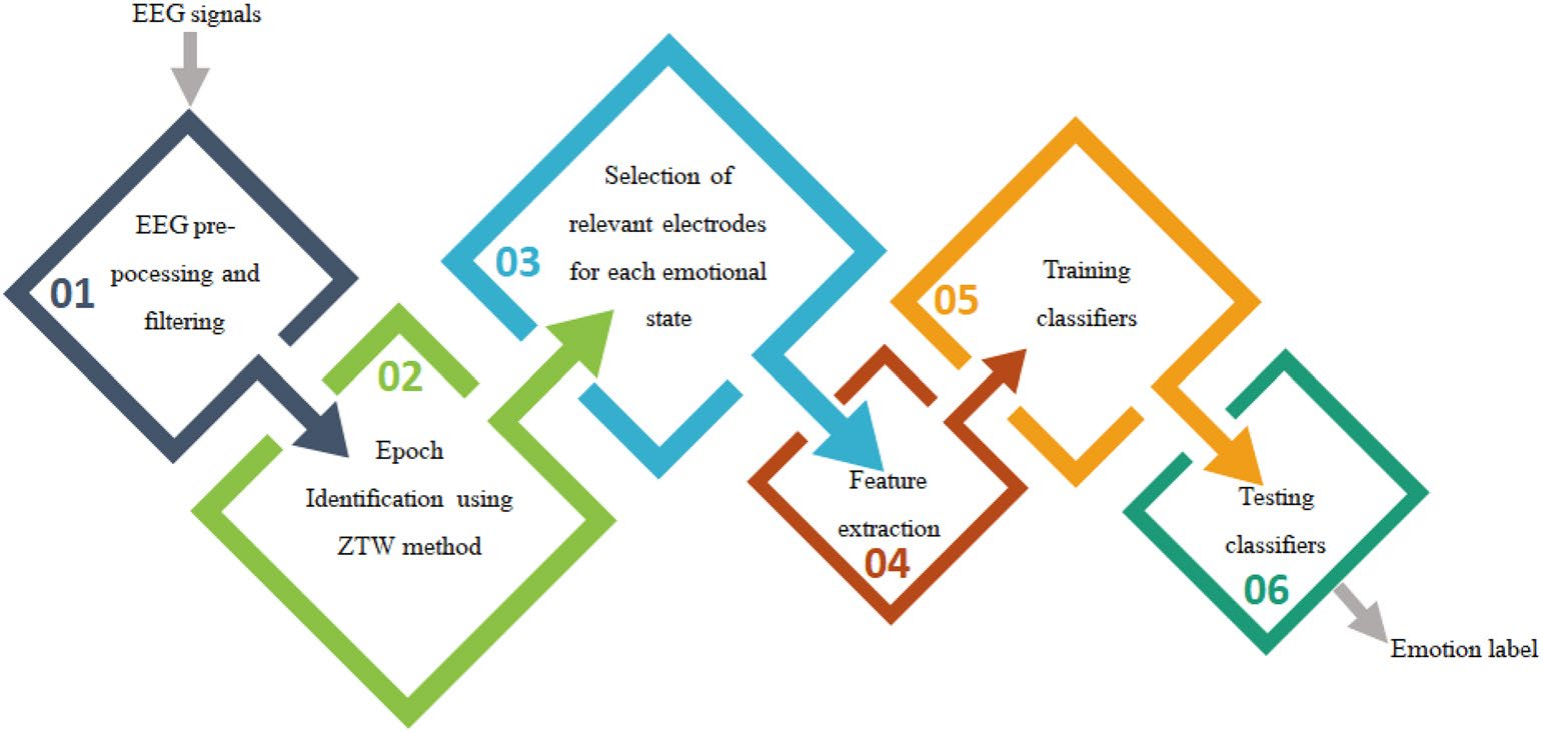
Schematic block diagram of the proposed method.

EEG signals reflect the electrical activity of the brain over time. They contain information about the control of the entire human body. We propose a new method to extract features from EEG sources based on the state of the brain of the individual under study. The proposed method identified the epoch in EEG signals with high resolution, then studied variation in brain activity in the selected electrodes. It determined the EEG channels that most likely showed the most significant changes in brain activity during emotions. These relevant electrodes were used as sources to extract features, and they change from one person to another, depending on their mental state. Each subject has a different group of electrodes chosen from the four EEG rhythms: alpha, beta, delta, and gamma. These sources were used to calculate the features used to recognize and classify nine emotions. The flow diagram of the proposed method is shown in Fig. 1.

### Annotations and terminology

In this section, we introduce the main terminology and annotations that are used in this paper. They are the key to understanding the proposed method. Let us define the following:

1. *F* is the set of frequency bands of brain waves. The frequency bands that we consider in this work are Theta $\theta$ (4 to 7 Hz), Alpha $\alpha$ (8 to 12 Hz), Beta $\beta$ (12 to 36 Hz), and Gamma $\gamma$ (36 to 42 Hz).
2. *Fs* is the sampling frequency of EEG signals.
3. An electrode, denoted as *e*, is an electrical conductor used to acquire brain signals. An electrode *e* has two main characteristics: (1) A label is the name of the electrode and indicates the cerebral region to which the electrode belongs, and (2) A serial number is a unique two-digit integer number within the cerebral region. If this number is odd, it implies that the electrode is in the left part of the brain lobe. Otherwise, it implies that the electrode is in the right part of the brain lobe.
4. *E* is the set of electrodes *e* of a cap.
5. An emotion, denoted as $\eta$, is the mental state that corresponds to human responses to environmental objects or events.
6. $\Psi$ is the set of emotional states. The emotional states that we considered in this work were happy, pleased, relaxed, excited, neutral, calm, distressed, miserable, and depressed.
7. A neutral state, denoted as $\eta _0$, was the mental state that corresponded to the neutral emotional state.
8. We defined $\varsigma$ as the set of human beings called subjects.
9. A trial, denoted as *t*, was a set of signals that were recorded using a set of electrodes *E*, and they corresponded to the brain activity of a given subject s during a given emotional state $\eta$.
10. $\Omega$ was the set of trials.
11. We defined the function $\tau$ as follows: $\tau$: $\Omega \rightarrow \varsigma \times \Psi$ $\tau (t_i)= (s,\eta )$. The $\tau$ function associates every trial to a given subject *s* and a given emotional state $\eta$.
12. $\tau ^{-1}: \varsigma \times \Psi \rightarrow P(\Omega )$ $\tau ^{-1}(s,\eta )=\{t_i \in \Omega$, such that $\tau (t_i) = (s, \eta )\} \subset \Omega$. It returns the subset of trials $t_i$ recorder from the subject *s* during the emotional state $\eta$.
13. We defined the function $\sigma$ as follows: $\sigma$: $\Psi \rightarrow P(\Omega )$ $\sigma (\eta )=\bigcup \limits _{si \in S} \tau ^{-1}(s_i,\eta )$ Given an emotional state $\eta$, this function returns the subset of trials that have been recorded from different subjects during the emotional state $\eta$.
14. We defined the function $\delta$ as follows: $\delta$: $\varsigma \rightarrow P(\Omega )$ $\delta (s)=\bigcup \limits _{\eta _i \in \Psi } \tau ^{-1}(s,\eta _i)$ Given a subject *s*, this function returns the subset of trials recorded from the subject *s* during different emotional states.
15. We defined $\varphi (s,\eta )$ as the set of trials recorded from *s* during the emotional state $\eta$. $\varphi (s,\eta )$ was defined as follows:
  1 $$\begin{aligned} \begin{aligned} \varphi : \varsigma \times \Psi \rightarrow P(\Omega )\\ \varphi (s,\eta )= \delta (s)\cap \sigma (\eta ) \end{aligned} \end{aligned}$$
16. We considered ${\mathscr {M}}_0^{s}=\varphi (s,\eta _0)$ as the set of trials recorded from s during emotions that corresponded to the neutral emotional state $\eta _0$.
17. The energy of a given electrode *e* in the frequency band *f* during the trial *t*, denoted *Energy*(*e*, *t*, *f*), was computed using two algorithms in this work: the discrete Fourier transformation (DFT) and NGD but in different contexts. It was measured according to the following expression (Eq. 2):
  2 $$\begin{aligned} \begin{aligned} Energy(e,t,f)=\sum _{k=\text {lower frequency of f}}^{\text {upper frequency of f}} kX(t,f)[e,k]\\ \end{aligned} \end{aligned}$$
  where *X* was computed according to Eq. (3) for the DFT representation and Eq. (4) for the NGD representation.

### Numerator group-delay

Recorded EEG signals are usually represented in a time domain. Advanced BCI systems map them from temporal representation (a time domain representation) into a spectral representation (a frequency domain representation) for accurate signal processing and to extract relevant features. DFT denoted by $\digamma$ is the most common technique to obtain the spectral representation of EEG signals. DFT was computed using the following equation.

3 $$\begin{aligned} \digamma [e,f]=\sum _{n=0}^{N-1}t[n,e]e^{\frac{-j 2\pi kn}{N}} \end{aligned}$$

**Figure 2:**
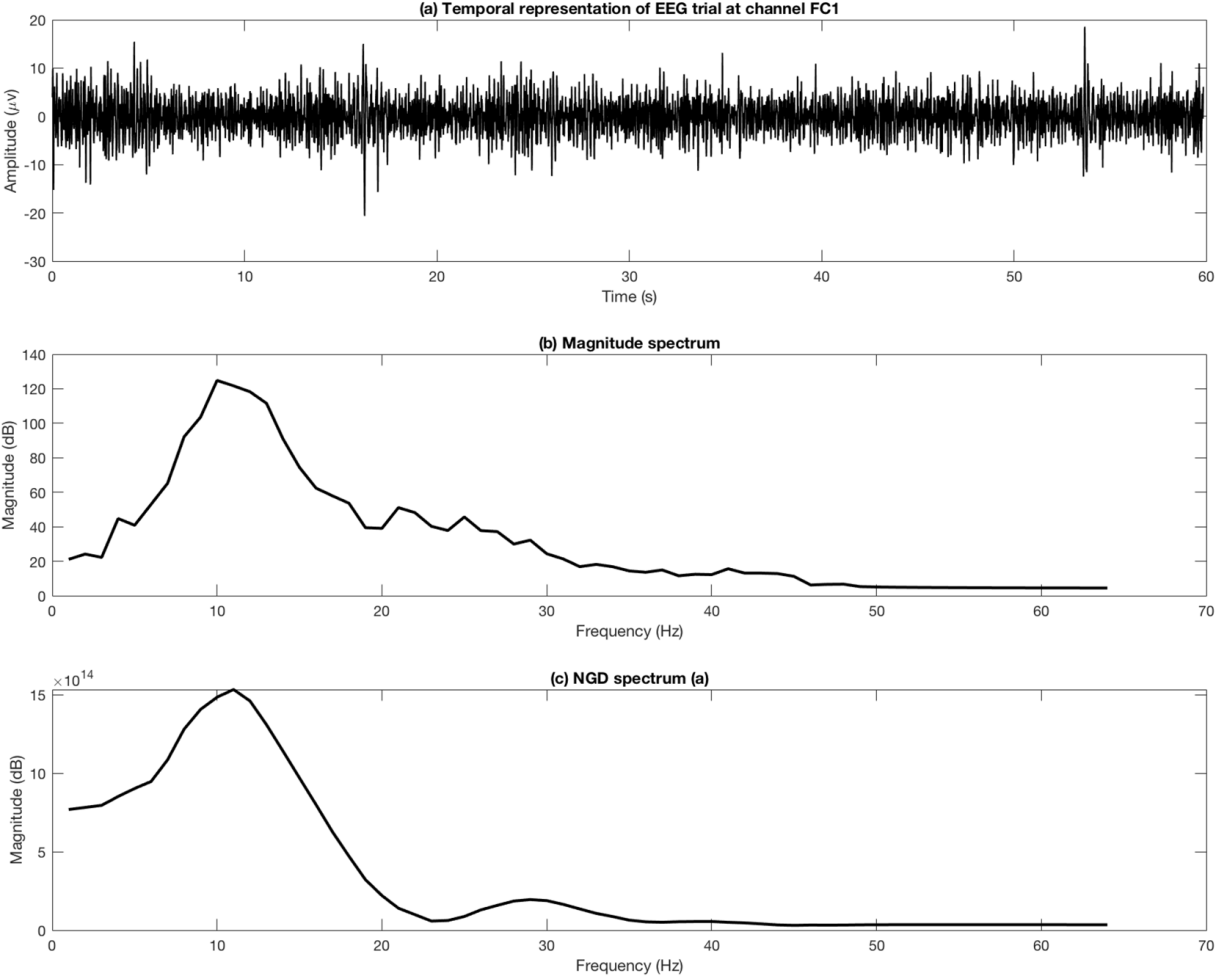
DFT and NGD representations of a given EEG trial at channel *FC*1.

The spectrum components or bins of the EEG signals were computed by keeping only the real part of $\digamma$ or by computing the absolute value of the real and imaginary parts of $\digamma$. Another function, called group-delay, also translated EEG signals from time domain to frequency domain. The group-delay function allowed the extraction of spectra with high-resolution properties and highlighted the formant features of the spectra. The group-delay function was computed according to the following equation (Eq. 3):

4 $$\begin{aligned} \varphi (t,e)=\frac{\mathfrak {R}(\digamma _\eta (e))\mathfrak {R}(\digamma _{\eta _n}(e))+\mathfrak {I}(\digamma _\eta (e))\mathfrak {I}(\digamma _{\eta _n}(e))}{\mathfrak {R}(\digamma _\eta (e))^2+\mathfrak {I}(\digamma _\eta (e))^2} \end{aligned}$$

where $\eta _n(n,e)=n\eta (n,e)$. The numerator of $\varphi$ was kept while maintaining the same accuracy of tracking the EEG formants to avoid division by zeros. Thus, the NGD function was designated. Figure 2a shows an example of an EEG trial corresponding to the excited emotional state at the electrode *FC*1. Figure 2b,c present the spectrum of the trial using the DFT and NGD methods, respectively. As shown, the NGD plot clearly kept the formant of the EEG trials at the same frequency bins where the fluctuations at the $\gamma$ band were removed. Thus, the identification of the active spectrum became more efficient.

### Zero-time windowing

**Figure 3:**
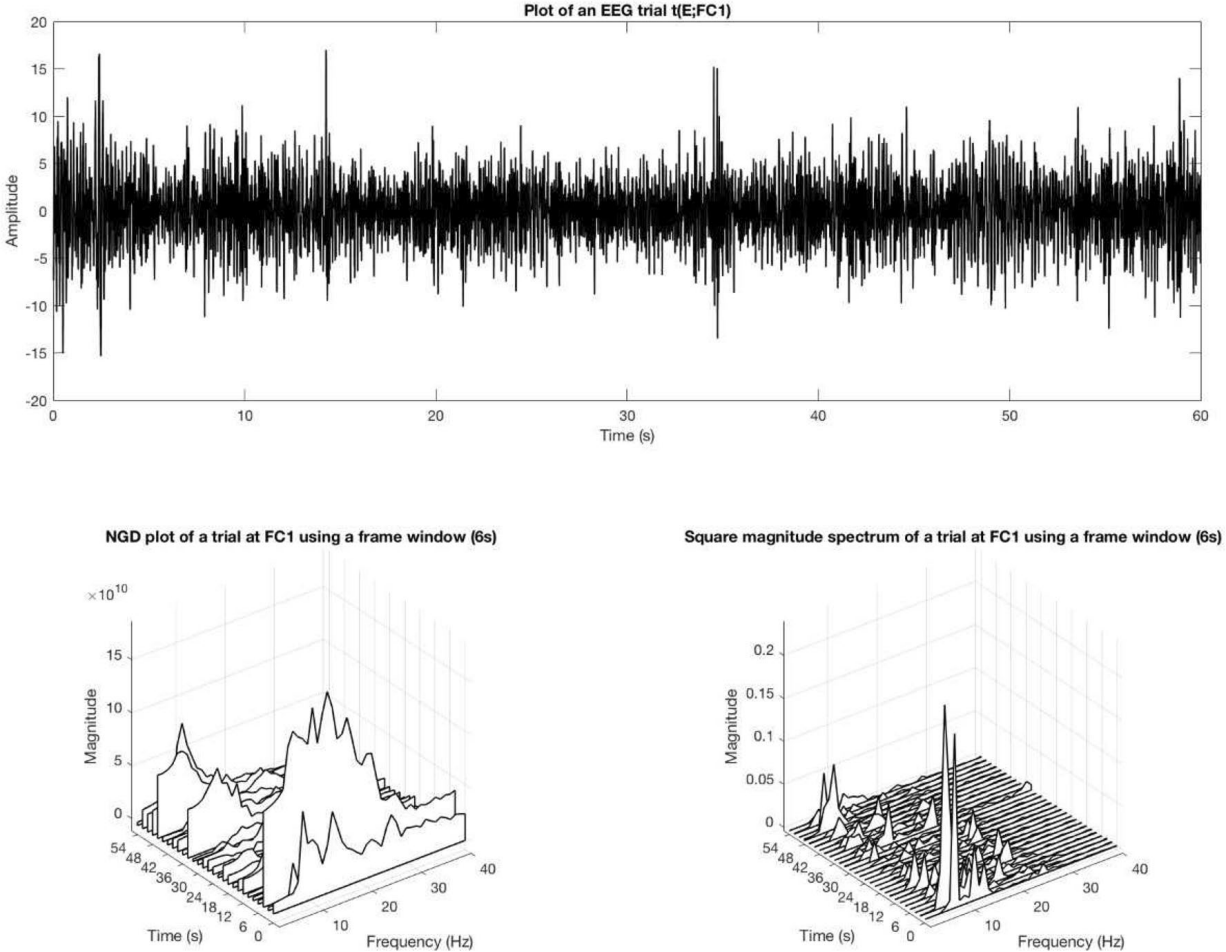
ZTW representations of EEG trial at channel *FC*1.

The ZTW approach was adopted to track and extract the spectral characteristics from short segments of EEG trials. The ZTW approach involves multiplying a short duration of each trial at each electrode with a window function similar in shape to the frequency response of a zero-frequency resonator^20^. The window function is given by:

$$\begin{aligned} \psi [n]=\left\{ \begin{array}{ll} 0,\; n=0 \\ \frac{1}{4\sin^2(\frac{\pi n}{2N})},\; n=1,2,...,F_l-1 \end{array} \right. \end{aligned}$$

where $F_l$ is the window length. The first value of the window $\psi [0]$ was initialized to zero to avoid a division by zero error and make the mean value of the spectrum of the windowed signal zero without altering the spectral peaks. Such a window function $\psi$ does not stop the discontinuities of the signal abruptness in the time domain, unlike any other window function^20^. Figure 3 shows the squared magnitude and the NGD plots of a trial $\eta (FC1)$ corresponding to an excited emotional state. For example, unlike the NGD plots, the squared magnitude plot shows an active rhythmic activity at the 3rd slot of the trial, where the brain activity in the other epochs are almost visually the same.

### ZTW-based epoch selection

An epoch reflects the maximum excitation of EEG signals during an emotional period. Detecting it is a challenge because of the variation in noise, mental tasks, eye movements, and the emotional state. Epoch detection has a significant role in improving the quality of the features of emotion recognition. We applied the ZTW-based epoch selection (ZTWBES) algorithm to select the significant epoch in every frequency band. ZTWBES consists of decomposing every trial into a collection of short segment EEG signals and identifying the frame location that corresponds to the epoch. We noticed that the epoch location changed from one subject to another.

**Figure 4:**
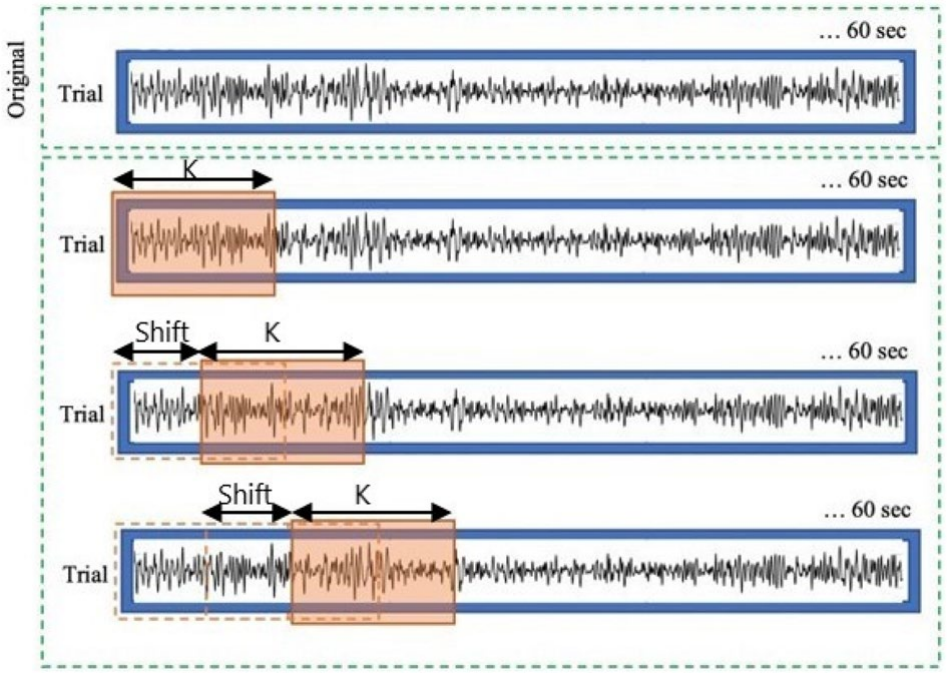
Frame windowing.

The ZTWBES is summarized below:

1. We defined $\kappa$ as the frame length of the epoch.
2. As depicted in Fig. 4 , we defined *Shift* as the time that separates the beginning and the start of two successive frames of the same EEG trial.
3. For every subject *s* of $\varsigma$, steps 4 to 18 were repeated.
4. For every EEG channel *e* of *E*, steps 5 to 13 were repeated.
5. We calculated the average energy, denoted $Average\_Energy\_\eta _0[e]$, of the brain activity of *s* in the channel *e* during the neutral emotional state $\eta _0$ for all frequency bands $f \in F$. The average energy was calculated according to the following expression:
  5 $$\begin{aligned} \begin{aligned} Average\_Energy\_\eta _0[e]=\frac{\sum _{t_j \in {\mathscr {M}}_0^{s}} Energy(e,t_j,[\theta .. \gamma ]) }{\parallel {\mathscr {M}}_0^{s} \parallel } =\frac{\sum _{t_j \in \varphi (s,\eta _0)} Energy(e,t_j, [\theta .. \gamma ]) }{\parallel \varphi (s,\eta _0) \parallel } \end{aligned} \end{aligned}$$
  where $Energy(e,t_j,[Theta.. Gamma])$ calculates the energy of the electrode *e* during the trial $t_j$ with respect to the frequency bands $\theta$, $\alpha$, $\beta$ and $\gamma$ using the NGD function.
6. For every trial *t* of $\delta (s)$, steps 7 to 12 were repeated.
7. The trial *t* was decomposed into a group of *n* frame instances, denoted *instF*, where *n* was calculated as follows: *n* = (($size\ of\ the\ trial \ t$ − $\kappa$)/*Shift*) + 1. A frame instance *instF*[*k*], where *k* = 1,2,..,*n*, starts at ((${k-1})\times {Shift}$) second and ends at (((*k* − 1)$\times$ *Shift*) + $\kappa$) second. For example, if $\kappa$ = 6 and *Shift* = 2, the first frame instance starts at 0*s* and ends at 6 s. The second frame instance starts at 2*s* and ends at 8 s, etc.
8. For every frame instance *instF*[*k*], where *k* = 1,2,..,*n*, steps 9 to 11 were repeated.
9. Apply the NGD function on *instF*[*k*] to obtain a spectrum *spectrum*[*k*] with high-resolution properties of *instF*[*k*].
10. *spectrum*[*k*] was used to calculate the energy $Energy(e, spectrum[k], [\theta .. \gamma ])$ in the electrode *e* during the frame instance *instF*[*k*] for the frequency bands $\theta$, $\alpha$, $\beta$ and $\gamma$. It was measured using the following expression (Eq. 6):
  6 $$\begin{aligned} \begin{aligned} Energy(e, spectrum[k], [\theta .. \gamma ]) = \sum _{f=\theta }^{\gamma } Energy(e, spectrum[k],f) = \sum _{f=\theta }^{\gamma } \sum _{k=\text {lower frequency of f}}^{\text {upper frequency of f }} k \times spectrum[k][e,f]\\ \end{aligned} \end{aligned}$$
11. Variation in the brain activity in the electrode *e* was calculated during the frame instance *instF*[*k*] compared with $Average\_Energy\_\eta _0[e]$. It was calculated using the following expression:
  7 $$\begin{aligned} \begin{aligned} variation =\left|\frac{ Energy(e,spectrum[k],[\theta .. \gamma ])- Average\_Energy\_\eta _0[e] }{Average\_Energy\_\eta _0[e]} \right|\end{aligned} \end{aligned}$$
  For accurate epoch detection, we calculated the variation in energy as an absolute value because brain activity may increase or decrease during emotional periods compared with brain activity during a neutral emotional state.
12. Tag the location *k* of the frame instance *instF*[*k*] for which the corresponding spectrum *spectrum*[*k*] has shown the maximum energy variation in the electrode *e* for the trial *t*.
13. Use the plurality voting algorithm to identify the location *k* that was tagged by the electrode *e* more than any other location. The elected location was considered as the potential candidate proposed by the electrode *e* for the next voting step.
14. Use the majority voting algorithm to select the frame instance location, denoted $winner\_location$, which was elected in step 13 by more than 50$\%$ of the electrodes.
15. Select the EEG channels with a vote that matches with the winner location $winner\_location$. We called *Elite* the set of selected electrodes.
16. *Elite*[*s*] was considered the group of electrodes chosen by the ZTWBES for the subject *s*.
17. *Epoch*[*s*] was considered the location of the epoch identified by the ZTWBES for the subject *s*.

This process was the first step in refining electrodes. As previously mentioned, we aimed to choose the best electrodes to enhance the quality of the features vector.

### Identification of relevant electrodes

A study in neuroscience published in 2016^7^, using functional magnetic resonance imaging (fMRI) scans of brain activity during different emotional sates, showed that the activity was unique for each emotion. Indeed, during each emotion, some of the brain lobes showed evident activity, while some did not. Based on this study and the assumption that the folding of the cortex differs between any two people, even monozygotic twins, our objective was to determine for every subject *s* which electrodes were relevant to every emotional state $\eta$ in every frequency band *f*.

For a given subject *s*, the ZTWBES algorithm identified the location *k* of the epoch and determined the set of electrodes, denoted *Elite*[*s*], which considered that the epoch was detected at location *k*. These electrodes were processed to determine their relevance to emotions. They were analyzed to determine, for every subject *s*, which ones were relevant to each emotion $\eta$ in every frequency band $f \in F$.

#### **Definition**

We considered a subject *s*, an emotional state $\eta$, and a frequency band *f*. The electrode *e* was relevant to the emotional state $\eta$ for the frequency band *f*, if the probability of obtaining significant brain activity changes in *e* with respect to *f* when the emotional state $\eta$ occurs, denoted $\rho (s,\eta ,f,e)$, exceeds a given threshold called $\alpha \_threshold$. The probability $\rho (s,\eta ,f,e)$ was calculated as follows (Eq. 8):

8 $$\begin{aligned} \begin{aligned} \rho : \varsigma \times \Psi \times F \times E \rightarrow \left[ 0,1 \right] \\ \rho (s,\eta ,f,e)=\frac{\parallel \gamma (s,\eta ,f,e) \parallel }{\parallel \varphi (s,\eta ) \parallel } \end{aligned} \end{aligned}$$

Here

- $\parallel \varphi (s,\eta ) \parallel$ and $\parallel \gamma (s,\eta ,f,e) \parallel$ were the cardinality of $\varphi (s,\eta )$ and $\gamma (s,\eta ,f,e)$, respectively.
- $\gamma (s,\eta ,f,e)$ was the subset of $\varphi (s,\eta )$, where the variations in the frequency band *f* of the brain activity of the subject *s* in the electrode *e* during the emotional state $\eta$ were significant. We considered that changes in the brain activity of a given subject *s*, during an emotional state $\eta$ in the electrode *e* with respect to the frequency band *f* were significant if brain activity changes/variations in *e* exceeded a given threshold called $\beta \_threshold$. Hence, $\gamma (s,\eta ,f,e)$ was defined according to the following expression: $\gamma : \varsigma \times \Psi \times F \times E \rightarrow P(\Omega )$
  9 $$\begin{aligned} \begin{aligned} & \gamma (s,\eta ,f,e)= \{t_i \in \varphi (s,\eta )\; \text {such that} \\ & \quad \quad |\Delta (e,t_i,Ref\_Energy(e,{\mathscr {M}}_0^{s},f),f)| \geqslant \beta '\_threshold \} \end{aligned} \end{aligned}$$
  We notice that in Eq. (9), the absolute value of brain activity changes during an emotional state $\eta$ is considered because the cerebral activity may increase or decrease during the emotional state $\eta$ compared with the Neutral state $\eta _0$. Variations in the brain activity were defined as the percentage of decrease or increase in energy in relation to a reference energy. The reference energy which was considered in this study is the average energy during the Neutral emotional state $\eta _0$. Hence, changes in brain activity of the subject *s* in the electrode *e* during the emotional state $\eta$ with respect to the frequency band *f* were calculated according to the following expression:
  10 $$\begin{aligned} \begin{aligned} \Delta (e,t_i,Ref\_Energy(e,{\mathscr {M}}_0^{s},f),f)=\frac{ Energy(e,t_i,f)- Ref\_Energy(e,{\mathscr {M}}_0^{s},f) }{Ref\_Energy(e,{\mathscr {M}}_0^{s},f)} \end{aligned} \end{aligned}$$
  where
  11 $$\begin{aligned} \begin{aligned} Ref\_Energy(e,{\mathscr {M}}_0^{s},f)=\frac{\sum _{t_j \in {\mathscr {M}}_0^{s}} Energy(e,t_j,f) }{\parallel {\mathscr {M}}_0^{s} \parallel } =\frac{\sum _{t_j \in \varphi (s,\eta _0)} Energy(e,t_j,f) }{\parallel \varphi (s,\eta _0) \parallel } \end{aligned} \end{aligned}$$

As such, we defined an electrode selector, denoted $\Gamma$, which determined for a given subject *s*, in a given frequency band *f*, the set of electrodes that are relevant to a given emotional state $\eta$. The selector $\Gamma$ was defined as follows (Eq. 12):

12 $$\begin{aligned} \begin{aligned} & \Gamma : \varsigma \times \Psi \times F \rightarrow P(E) \\ & \quad \quad \Gamma (s,\eta ,f)= \{e_i \in Elite[s], \text {such that } \rho (s,\eta ,f,e_i)\geqslant \alpha \_threshold \} \end{aligned} \end{aligned}$$

### Classification

The classification problem that we are concerned with in this paper involves recognizing the current emotion from eight distinct emotions. Every subject *s* had a feature vector for every emotional state $\eta$ in every frequency band *f* of *F*. A feature vector input set consisted of the brain activity changes in the corresponding relevant electrodes determined by $\Gamma (s,\eta ,f)$. We adopted two classification algorithms: QDC and RNN. Using QDC, we adopted a two-step classification strategy. We adopted two different classification schemes using RNN.

#### Quadratic classifier (QDC)

##### Overview of the quadratic classifier (QDC)

A quadratic classifier is the general version of the linear classifier. It uses a quadratic decision surface to separate between the classes of the problem. The QDC is one of the famous Bayesian parametric classifiers in multiple applications. It is based on the theory that the class-distinct densities of the feature vector are multivariate Gaussian. As a result, it works by approximating the variance-covariance matrix variable for each class^21^. The quadratic function is as follows (Eq. 13)^22^:

13 $$\begin{aligned} \begin{aligned} \delta (x)=-\frac{1}{2}\log \mid \scriptstyle \sum _{k}\mid - \frac{1}{2}(x-\mu _k)^T\scriptstyle \sum _{k}^{-1}(x-\mu _k)+\log \phi _k \end{aligned} \end{aligned}$$

And the classification rule is asAnd the classification rule is as in the linear classifer (Eq. 14)

14 $$\begin{aligned} \begin{aligned} {\hat{G}}(x) = \arg \max _k\delta _k(x) \end{aligned} \end{aligned}$$

We used QDC with 10-fold cross-validation and Prtools library for data representation with QDC.

##### Classification strategy using QDC

**Figure 5:**
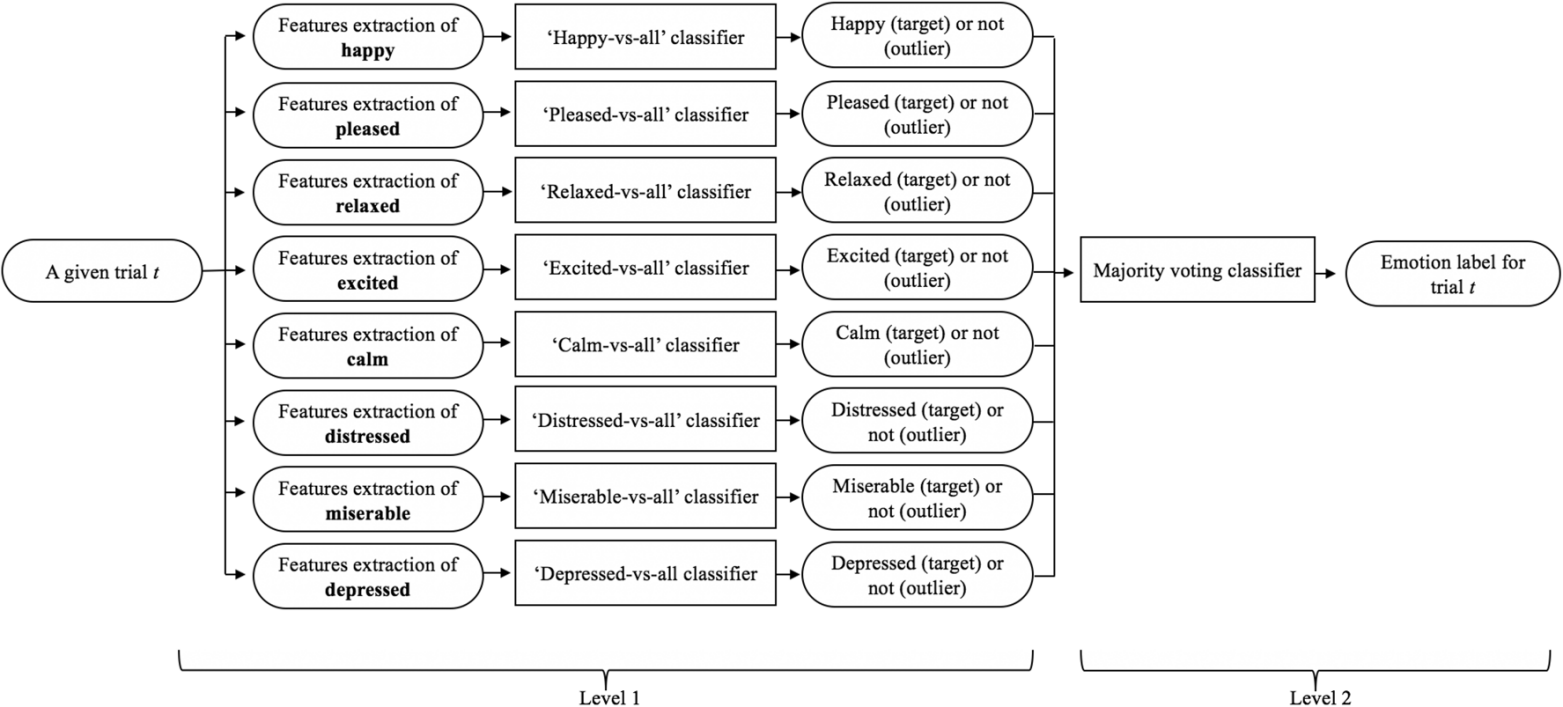
Schematic of classification.

The classification problem that we addressed in this paper is a multi-class classification problem and it was solved in two phases. As depicted in Fig. 5, the first phase aimed to predict which different emotional states a given trial could belong to. For this purpose, we defined eight $one-vs-all$ QDC based classifiers: a classifier for every emotional state. The *Neutral* emotional state was excluded because it was the reference state. These $one-vs-all$ QDC based classifiers were trained using a set of trials of the corresponding emotional state, called the target emotion, and certain trails of the other emotions, called outliers. Every classifier extracted the features vector from the electrodes that were relevant to its corresponding target emotional state. Given a trail *t*, every classifier calculated, in every frequency band *f*, changes in brain activity in the electrodes that are relevant to the corresponding target emotion. Based on these changes, every classifier predicted whether the given trial *t* belonged to its corresponding target emotional state or not. As such, we obtained eight intermediary decisions regarding the same trail *t*.

The second phase aimed to infer the exact emotional state that the trial *t* most probably belongs to. For this purpose we adopted a majority voting algorithm. If the majority of the $one-vs-all$ classifiers vote *target* in phase one, this meant that there was an error. If the majority of the $one-vs-all$ classifiers vote *outlier* in phase one, we considered that the trial *t* corresponded to the target of the $one-vs-all$ classifier, which classified the trial *t* as a target and obtained the highest accuracy rate during the training phase among all $one-vs-all$ classifiers, which classified the trial *t* as a target. If all $one-vs-all$ classifiers voted *outlier* in phase one, this meant that the trail corresponded to the *Neutral* emotional state.

#### Classification using RNN

##### Overview of RNN

**Figure 6:**
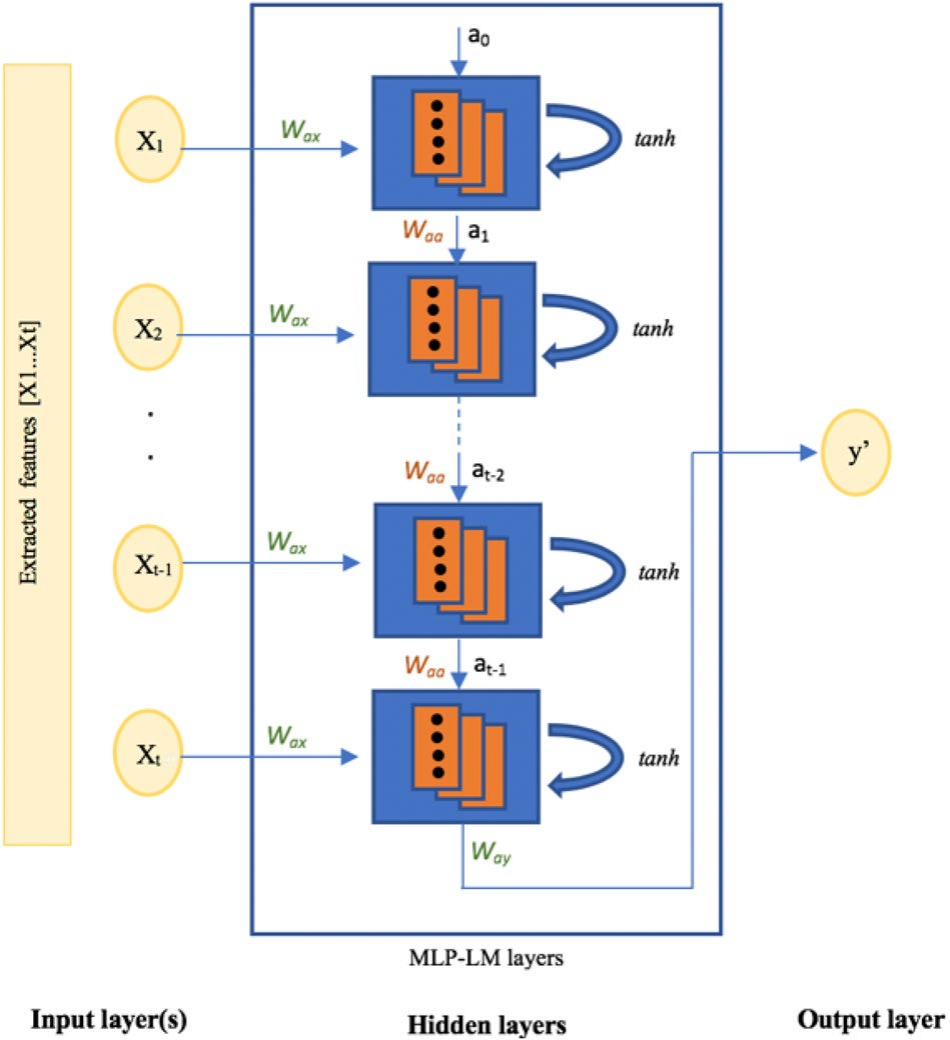
Recurrent neural network structure.

The adaptive method of choosing EEG electrodes leads to the selection of different sizes of a feature vector from person to another. Therefore, using an RNN in this case is suitable, especially for fast and effective processing in this neural network. The RNN is a deep learning neural network that processes sequential data on a time axis. It is an improvement of the convolutional neural network, which is limited by a fixed number of inputs and outputs and by fixed data flow in the hidden layer. In the RNN, there is a flexibility in the number of inputs and outputs (one-to-one, one-to-many, and many-to-many). The structure of an RNN as an NN consists of an input layer, hidden layers, and output layers. The input and output can be a sequence of data. Each RNN layer is a combination of a number of hidden layers with the same weight and bias (Fig. 6).

The activation function of RNN is $g(t)=tanh(t)$, and the formula for inputs is $a_t=g(W_{aa}a_{t-1}+W_{ax}X_t+b)$, where $a_t$ is the current state of the RNN current layer, $a_{t-1}$ is the previous state of the previous layer, and *X* is the input for that RNN layer. $W_{aa}$ and $W_{ax}$ are weight vectors, and *b* is the bias factor. An RNN can be used with different error minimization methods. We used the Levenberg–Marquardt (LM) algorithm, which is an algorithm to solve nonlinear least squares problems^23^. The LM reduces squared errors by changing the parameters with the steepest-descent direction in the same way as in the gradient descent method. The aim of LM is to find the best perturbation h to parameter p to minimize the error value represented by $X^2$ in this formula:

15 $$\begin{aligned} \begin{aligned} X^2=\sum _{i=1}^{m} \left[\frac{y(t_i)-{\hat{y}}(t_i;p)}{\sigma _{yi}} \right]^2=(y-{\hat{y}}(p))^TW(y-{\hat{y}}(p))=y^TWy-2y^TW{\hat{y}}+{\hat{y}}^TW{\hat{y}} \end{aligned} \end{aligned}$$

where $X^2$ is the sum of the weighted square errors of the main $y(t_i)$ data and the curve fit function ${\hat{y}}t_i;p)$, $\sigma _{yi}$ is the measurement error of the measurement $y(t_i)$, and finally, *W* is the weight matrix, calculated by $W_{ii}=\frac{1}{\sigma ^2_{yi}}$. As we mentioned above, LM is attempting to find the best perturbation *h* to parameter *p* as below:

16 $$\begin{aligned} \begin{aligned} \left[ J^TWJ+\lambda diag(J^TWJ)\right] h_{LM}=J^TW(y-\hat{(}y)); \text { where } {\hat{y}}(p+h)\cong {\hat{y}}(p)+\frac{\partial {\hat{y}}}{\partial p}h={\hat{y}}+Jh \end{aligned} \end{aligned}$$

and $\lambda diag$ is the damping parameter that increases and decreases depending on the approximation of $X^2(p+h_{LM}) \geqslant X^2(p)$ in each iteration.

##### Schema 1: ensemble of one-vs-all RNN-based classifiers

The same classification strategy, described in Fig. 5, was adopted again expecting that we changed every $one-vs-all$ QDC based classifier with a $one-vs-all$ RNN-based classifier. The details of $one-vs-all$ RNN-based classifiers were obtained through various experiments and search for the best structure. The best accuracy results were gathered from the following structure:

- The network comprised a number of input layers equal to the number of the electrodes that were relevant to the corresponding target emotion. The size of the feature vector was different from one subject to another, depending on the brain activity of the subject.
- There were three hidden layers to receive each feature vector component. Each layer was an MLP layer with a loop inside and delay factors. They contained three neurons to receive input components, and then the activation function *tanh* was applied.
- The classification system we propose is static: we set the delay between inputs to empty because we did not focus on any dependencies in the emotions between trials. A DEAP dataset used independent videos in the experiments. Thus, there was no need to set any input, internal, or output delays.
- The output layer was related to the scheme of the classifier. The output was a rational value in the range [− 0.1,1.9], which we normalized to be 0 or 1, reflecting the labels target or outlier.

##### Schema 2: all-together RNN-based classifier

**Figure 7:**
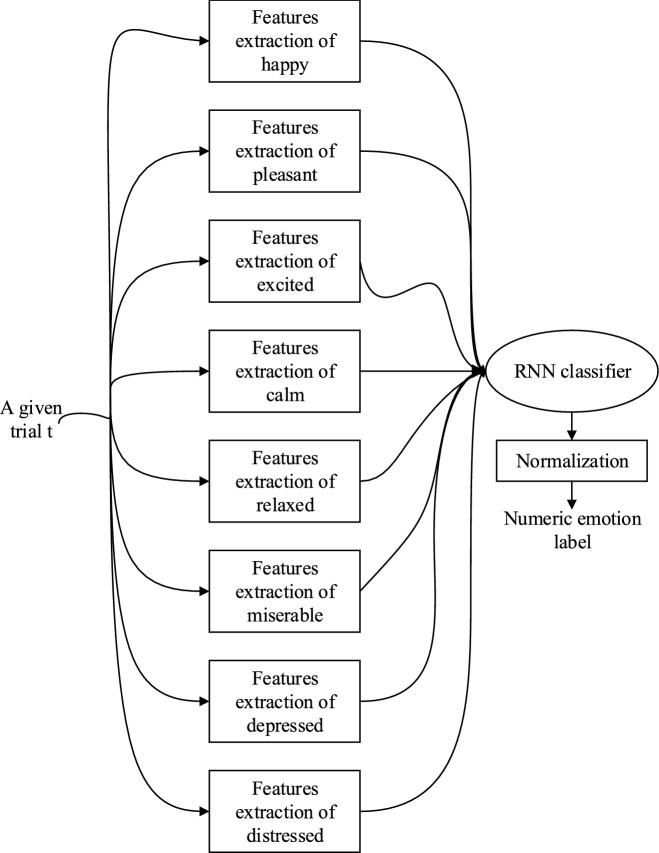
Schematic block diagram of the proposed all-together RNN-based classifier.

As depicted in Fig. 7, this approach adopted a single $all-together$ RNN-based classifier to directly predict emotion and provide a distinctive label. This schema combined all the feature variables of all classes and then tried to find the correct class. It is a complex scheme and it requires an optimization formulation to optimize all variables^7^. The feature vector contained all relevant electrodes of all emotions in all frequency bands for every subject. An RNN can handle the dimension of the feature vector although it takes more time than the previous approach. As we will show in the discussion section, some emotions are easy to predict using the adaptive method and some are not.

## Performance evaluation of the proposed method

### Overview of the DEAP dataset

DEAP dataset is one of the famous datasets in the field of emotion recognition based on EEG signals. The state of 32 subjects was recorded while they watched music videos^24^. Every subject was recorded during 40 videos (40 trials) using 40 channels (32 were EEG electrodes and the rest were peripheral channels), which recorded at a sampling rate of 512 Hz then down sampled to 128 Hz. Each subject had an array of video/trial and each trial had four labels: arousal, valence, dominance, and liking/disliking. The subjects also rated the valence, arousal, dominance, and like/dislike using a self-assessment questionnaire^24^. We mapped the dimensions to nine distinct emotions related to the DEAP recordings: happy, pleasant, relaxed, calm, excited, neutral, distressed, miserable, and depressed^24^.

### EEG signals preprocessing and configuration settings

The nature of the EEG requires accurate prepocessing and removal of noise/artifacts. In the DEAP dataset, the electrooculogram (EOG) data (eye-generated artifacts) were removed and a high pass filter was applied. In addition, we applied a band-pass filter to obtain alpha, beta, theta, and gamma signals for each trial.

During the EEG channel selection process, we applied ZTW and NGD with a frame length of 15 s and a shift of 15 s. We set the $\alpha \_threshold$ and $\beta \_threshold$ to 0.5 to select relevant electrodes of every emotion in every frequency band.

Finally, in the classification stage, we tested different values and settings to find the best performance and accuracy for the RNN classifier. The RNN consists of input layers of the same size as the chosen electrodes, three hidden layers, and one output layer to produce the emotion label. There were five iterations with weight and biases between [− 0.5,+ 0.5].

**Figure 8:**
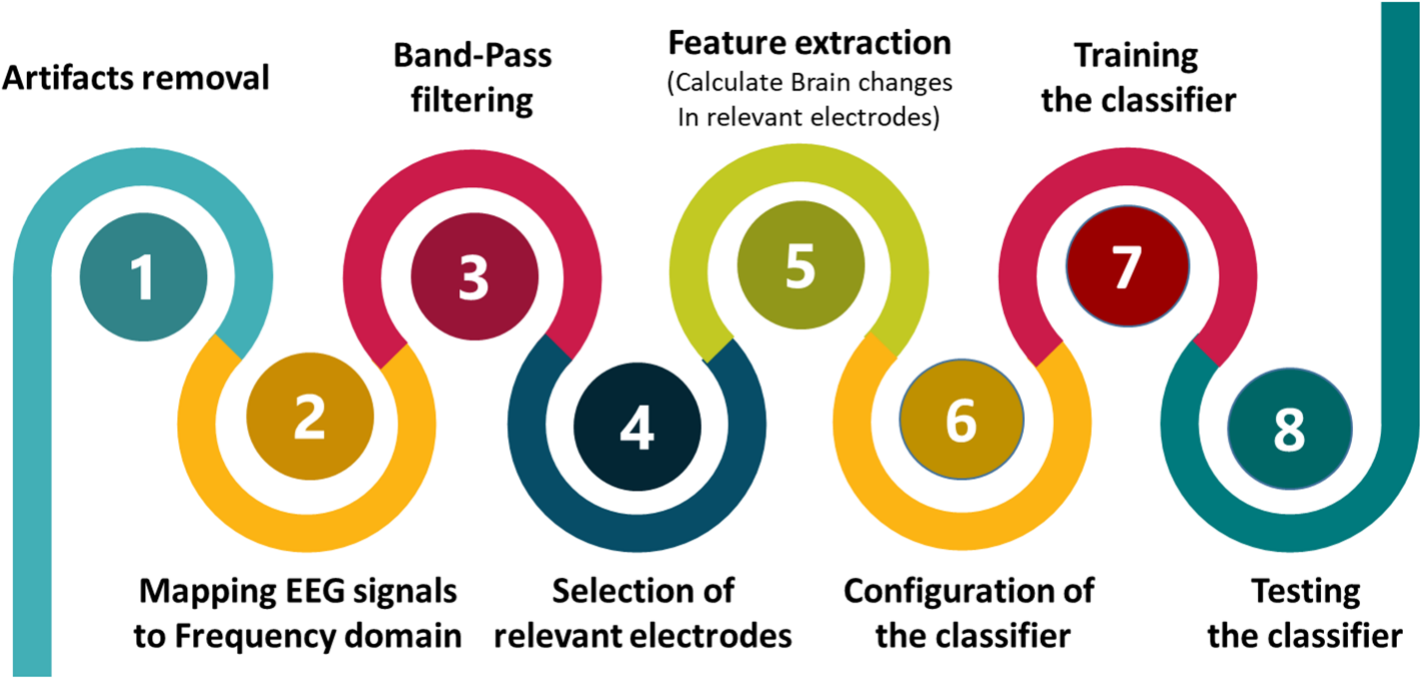
Experiments diagram: without epoch identification scenario.

**Figure 9:**
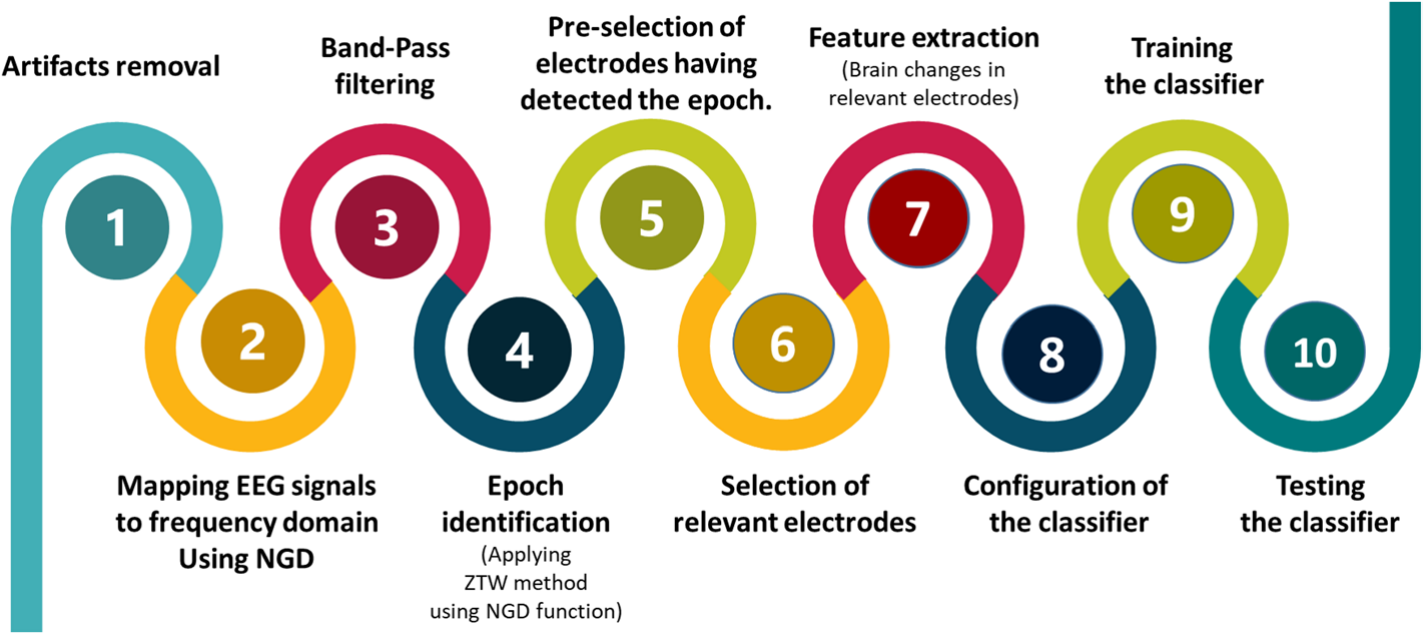
Experiments diagram of the proposed method.

Every classification approach, described above, was tested and evaluated using the following three experiments:

- Experiment 1, which was called $DFT_{Exp}$, is depicted in Fig. 8. This experiment aimed to proof that the selection process of relevant electrodes, using the DFT function, contributes to an accurate emotion recognition. The experiment started by applying a high pass filter to remove noise and artifacts (step 1) from EEG signals. Then, EEG signals were mapped to the frequency domain (step 2) using the DFT function (Eq. 3). The obtained signals were filtered using a band-pass filter (step 3) to keep only signals in the alpha, beta, theta, and gamma bands. The electrodes selector $\Gamma (s,\eta ,f)$ was applied on the filtered signals to determine for every subject *s*, in every frequency band *f*, the set of electrodes that are relevant to every emotional state $\eta$ (step 4). Brain changes in the selected electrodes (step 5) were used as features to train (step 7) and test the classifier (step 8). The configuration and settings of the classifier were specified and reviewed again and again in step 6 until the best performance was reached.
- Experiment 2, which was called $NGD_{Exp}$, had the same steps as $DFT_{Exp}$ except that the mapping of EEG signals from the time domain to the frequency domain was conducted using the NGD function (Eq.  4). This experiment aimed to show that the spectral information obtained using the NGD function instead of DFT function improves the accuracy of the system.
- Experiment 3, which was called *ProposedMethod*, is depicted in Fig. 9. This experiment aimed to demonstrate the efficiency of the proposed method to increase the accuracy of the entire emotion detection process. After removing noise and artifacts using a high pass filter (step 1), EEG signals were mapped to frequency domain (step 2) using the NGD function (Eq.  4), and then a band-pass filter was applied (step 3) to retain only signals in the alpha, beta, theta, and gamma bands. The filtered signals were processed using the ZTWBES algorithm to identify, for every subject *s*, the location of the epoch denoted *Epoch*[*s*] (step 4), and then to select the electrodes, denoted *Elite*[*s*], having identified the calculated epoch location (step 5). The electrodes selected in step 5, *Elite*[*s*], were processed in the next step using the electrodes selector $\Gamma (s,\eta ,f)$ to determine for every subject *s*, in every frequency band *f*, the electrodes that are relevant to every emotional state $\eta$ (step 6). Brain changes in the selected electrodes (step 7) were used as features to train (step 9) and test the classifier (step 10). Then, the configuration and settings of the classifier were specified and reviewed again and again in step 8 until obtaining the best performance.

The model was implemented using MATLAB with some efficient toolboxes, such as EEGLAB, prtools, Data description MATLAB toolbox (dd tools), machine learning toolbox, and deep learning toolbox. The best simulation parameters used are listed in Table 1.

**Table 1:** Parameters used in simulations.

Process	Parameter	Value
ZTW based epoch selection	$\kappa$	15
	*shift*	15
Identification of relevant electrodes	$\alpha \_threshold$	> 0.5
	$\beta \_threshold$	> 0.5
RNN	Input layers	$\mid \Gamma \mid$
	Hidden layers	3
	Number of nodes	3
	Iterations	5
	Weights	[− 0.5, + 0.5]
	Bias	[− 0.5, + 0.5]
	Adapt damping factor of LM	10
	Damping factor of LM	3
	Delay factor	0
	Error E (cost function)	$110^-10$

### Results

Table 2 represents a sample of relevant electrodes. The sample shows the relevant electrodes for an arbitrarily subject for every emotional state in every frequency band. The group of electrodes identified using ZTWBES were listed in the first row of Table 1. These electrodes were processed to determine the relevant electrodes for every emotional state in every frequency band.

**Table 2:** Example of relevant electrodes after ZTW-based epoch selection for an arbitrary subject (in every frequency band).

Electrodes selected by the ZTWBES algorithm			Fp1	F3	F7	C3	T7	CP5	P3	O1	Fp2	F8	T8
Refining relevant electrode for each emotion (step II)	Alpha band	Happy	Fp1	–	F7	–	T7	CP5	–	O1	–	–	T8
		Pleasant	Fp1	F3	F7	–	T7	–	P3	O1	Fp2	F8	T8
		Relaxed	–	–	–	–	T7	CP5	–	–	–	F8	–
		Excited	Fp1	F3	F7	C3	T7	–	P3	O1	Fp2	F8	T8
		Calm	Fp1	–	–	–	T7	CP5	P3	–	–	–	–
		Distressed	Fp1	–	F7	–	T7	–	–	O1	–	F8	T8
		Miserable	–	–	F7	–	–	CP5	P3	O1	–	–	–
		Depressed	Fp1	–	F7	–	T7	CP5	–	O1	–	F8	T8
	Beta band	Happy	–	F3	–	C3	T7	–	–	O1	Fp2	F8	
		Pleasant	–	–	–	C3	–	–	–	–	–	F8	T8
		Relaxed	Fp1	F3	–	–	T7	–	P3	O1	Fp2	F8	–
		Excited	–	F3	F7	C3	T7	–	P3	O1	–	F8	–
		Calm	–	F3	F7	C3	–	CP5	–	–	–	F8	T8
		Distressed	–	F3	–	C3	–	CP5	–	O1	–	F8	T8
		Miserable	–	–	F7	C3	–	–	–	O1	–	F8	–
		Depressed	–	F3	–	C3	–	–	–	O1	Fp2	F8	T8
	Theta band	Happy	Fp1	–	–	–	–	–	–	–	–	F8	–
		Pleasant	–	–	–	C3	–	CP5	–	–	–	–	–
		Relaxed	Fp1	–	–	–	–	–	–	–	–	F8	–
		Excited	–	F3	F7	C3	–	–	–	–	Fp2	–	T8
		Calm	–	F3	–	–	–	–	–	–	Fp2	–	T8
		Distressed	Fp1	–	–	–	–	–	P3	O1	Fp2	F8	T8
		Miserable	Fp1	–	–	–	–	–	–	–	–	–	–
		Depressed	Fp1	–	–	–	–	CP5	–	–	–	–	–
	Gamma band	Happy	Fp1	F3	F7	C3	–	CP5	P3	O1	–	F8	T8
		Pleasant	Fp1	–	–	C3	–	CP5	–	–	–	F8	–
		Relaxed	Fp1	–	F7	C3	T7	CP5	P3	–	Fp2	–	T8
		Excited	Fp1	F3	F7	C3	T7	CP5	P3	O1	–	–	T8
		Calm	–	–	F7	–	T7	–	P3	O1	Fp2	–	T8
		Distressed	Fp1	F3	F7	C3	–	CP5	P3	O1	–	F8	T8
		Miserable	Fp1	F3	F7	C3	T7	–	P3	O1	Fp2	–	–
		Depressed	–	F3	F7	C3	T7	–	P3	O1	–	F8	T8

The original number of electrodes is 32 from DEAP datasetThe original number of electrodes is 32 from DEAP dataset.

**Figure 10:**
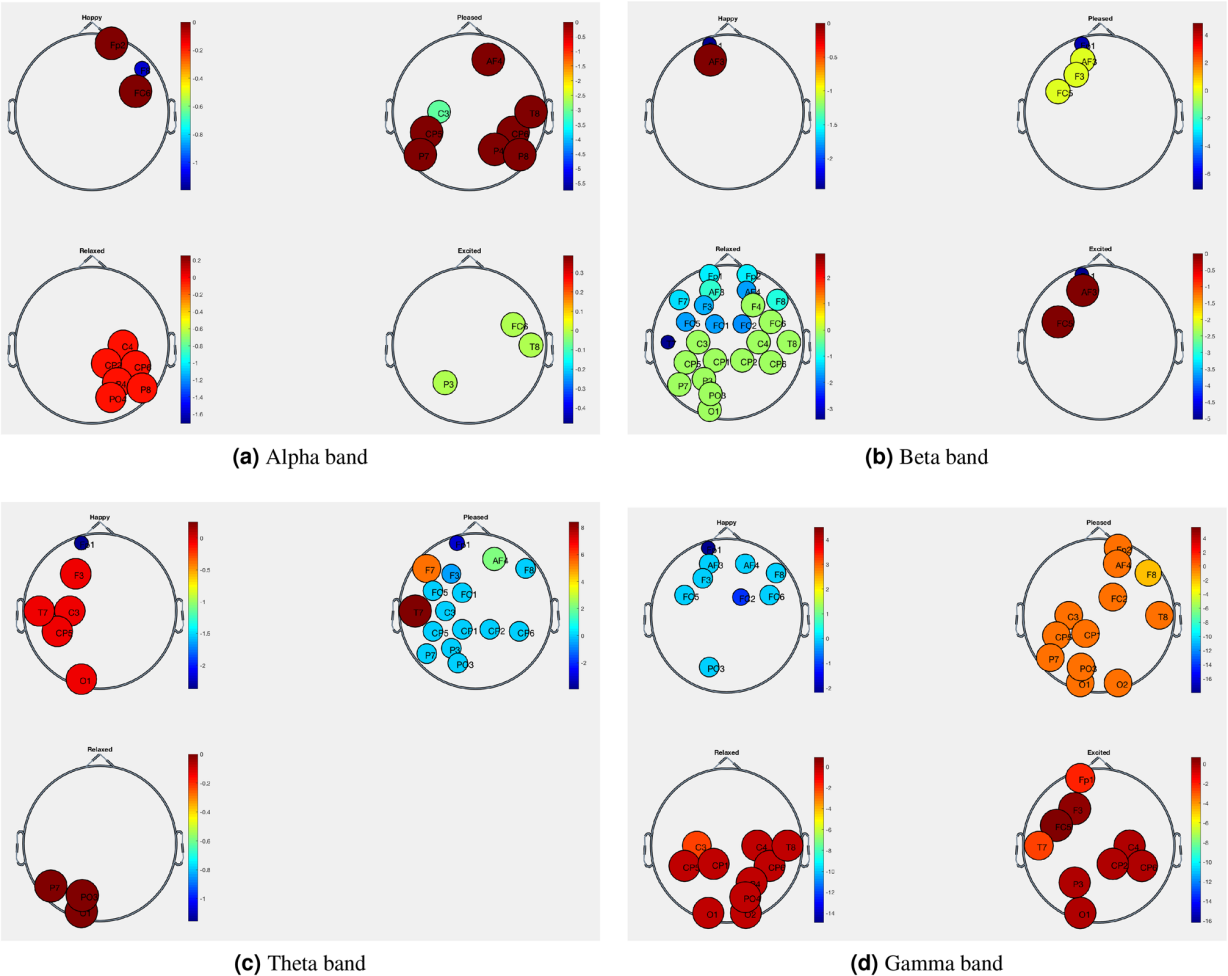
Significant electrodes pool for emotions: happy, pleased, relaxed, and excited in all the four bands for an arbitrary subject. The volume and color of each node represented the variation of the electrode activity during the emotion status.

As depicted in Fig. 10, our analysis of EEG signals showed that brain activity changed from one emotional state to another in the same frequency band. Moreover, brain activity changed from one frequency band to another during the same emotional state.

**Figure 11:**
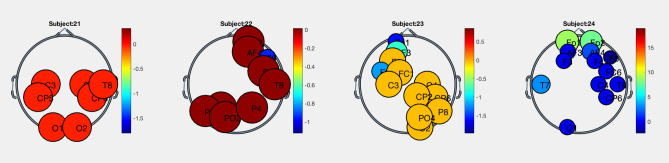
Significant electrodes pool for emotion excited in aplha band for four different subjects.

Besides, as illustrated in Fig. 11, the brain activity during the same emotional state changed, in the same frequency band, from one subject to another.

Table 3 shows the accuracy obtained using the different classification approaches, which we adopted in this work, in the different three experiments described above. Table 3 shows the average accuracy for every emotion (one-against-all level) and the final decision classification (majority voting level) for QDC and RNN-scheme1. It also shows the average emotion classification for each emotion label and the average of the RNN-scheme 2 for all the subjects in the study.

**Table 3:** Accuracy results (%) of the proposed method.

Emotion ($\eta$)	QDC			RNN (scheme 1)			RNN (scheme 2)		
	$DFT_{Exp}$	$NGD_{Exp}$	Proposed method	$DFT_{Exp}$	$NGD_{Exp}$	Proposed method	$DFT_{Exp}$	$NGD_{Exp}$	Proposed method
Happy	67.90	72.43	73.99	85.95	90.29	91.19	86.79	88.98	92.04
Pleased	81.69	86.68	88.48	80.82	89.44	88.04	89.67	87.46	90.83
Relaxed	79.63	84.11	85.67	88.02	91.47	92.74	72.70	74.49	70.87
Excited	71.92	74.40	78.05	91.15	95.29	94.25	72.25	57.05	67.42
Calm	84.20	89.94	92.63	79.09	87.32	86.21	87.75	83.99	88.88
Distressed	68.21	74.09	73.01	87.62	92.01	93.10	69.16	69.78	75.20
Miserable	71.79	76.17	82.99	83.99	91.38	92.12	85.41	96.08	98.79
Depressed	79.26	79.46	84.18	84.14	90.90	92.12	94.46	91.01	93.21
Neutral	N/A	N/A	N/A	N/A	N/A	N/A	87.10	93.44	93.49
AVG	75.57	79.66	82.37	85.10	91.01	91.22	82.81	82.48	85.64
Final decision	79.86	83.67	87.05	78.13	84.48	89.33	83.83	82.86	86.53

Moreover, Table 3 shows the overall average accuracy for all eight emotion classifiers in the QDC and RNN-scheme 1. This average indicates the average accuracy of classifying the emotion labels for the RNN-scheme 2. We would like to emphasize that the neutral emotional state did not have a standalone classifier using the QDC and RNN-scheme 1 because it was used as the reference (baseline) emotional state in this study. However, the final decision classifier (majority voting in QDC and RNN-scheme 1) could include the neutral state and the other eight emotions. Also, it was classified in the RNN-scheme 2.

The results show the efficiency of applying ZTW to select effective epochs of the EEG signals and the performance of the final classification stage was increased by + 3.38% and + 7.86% compared with $NGD_{Exp}$ and $DFT_{Exp}$ experiments, respectively. RNN is also an efficient addition to this work. It is shown that all performance metrics were increased using deep learning. It scores an average above 90% when used with NGD and ZTW. Again, applying ZTW improves the accuracy of the final decision level by + 4.85% and + 11.2% compared with $NGD_{Exp}$ and $DFT_{Exp}$ experiments, respectively in the RNN-scheme 1; and by + 2.83 and + 3.18 in RNN-scheme 2. We conclude that using NGD with ZTW for channel selection and deep learning to classify emotions is a competitive combination in this proposed method.

### Benchmarking and discussion

The results of the proposed method lead us to compare it with recent works in the field. We chose the studies that used the locationist model and worked on the DEAP dataset. We summarize the comparison in Table 4.

**Table 4:** Comparison between the proposed method and related works.

Method	Number of classes	Feature extraction algorithm	Number of electrodes	Classifiers	AVG accuracy(%)
^18^	3 emotions (anger, surprise, other)	Minimum redundancy maximum relevance (mRMR)	32	SVM-random forest	60
^14^	4 emotions (happy, sad, angry, and relaxed)	Time and frequency domain features	5	Decision tree algorithm	81.64
^15^	4 emotions (angry, sad, happy, and relaxed)	time domain features, frequency domain features and entropy	32	ANN	93.75
^13^	4 emotions	Probability distribution for wavelet packet coefficient	3	SVM	70.5
^19^	9 emotions	Spectral features	32	DBN	79.2
^8^	9 emotions	Fusing of 6 statistical features	32	SVM	81.87
Proposed method	9 emotions	ZTWBES	Adaptive	QDC	87.05
				RNN-scheme 1	89.33
				RNN-scheme 2	86.53

As shown in Table 4, the proposed method is highly competitive compared with existing studies of the emotion recognition problem. Additionally, deep learning algorithms enhanced the results of the proposed method.

## Conclusion

This paper presented a new approach for emotion recognition using EEG signals. The proposed approach consists of applying the ZTWBES algorithm to identify the epochs, pre-selecting the electrodes that successfully identified the epochs and for every emotional state, determining relevant electrodes in every frequency band. Brain activity changes compared with neutral emotional states were extracted as features. As such, unlike previous studies, the set of electrodes processed to extract relevant features for the distinct emotions changed in every frequency band from one emotion to another, and from one subject to another.

Three classification strategies were applied to measure the efficiency of the proposed method. Our findings showed that the proposed method was a competitive approach in this field and it outperformed that of previous studies. The QDC obtained an average accuracy of 82.37%, and the RNN reached an accuracy average of 91.22% and 85.64% during classification schemes 1 and 2, respectively. Compared with the best performance obtained by previous studies, the proposed method increased the accuracy of emotion recognition by 2.37%, 11.22%, and 5.64% using the QDC, RNN-scheme 1, and RNN-scheme 2, respectively.

Contrary to the image processing based approach, emotion detection using EEG signals requires multi-disciplinary skills including neuroscience, engineering, computer science and psychology. However, going beyond performances of current algorithms for emotion detection, requires further discovery in neuroscience and psychology or by applying a multi-modal approach that combines EEG-based emotion recognition models with image processing based approaches.
